# Potential neuroprotective and therapeutic agents and their mechanisms for irradiation‐induced brain injury

**DOI:** 10.1002/ibra.70011

**Published:** 2026-01-15

**Authors:** Seidu A. Richard, Vivian Kapio Abem, Sagor Kumar Roy

**Affiliations:** ^1^ Department of Biochemistry and Forensic Sciences, School of Chemical and Biochemical Sciences C. K. Tedam University of Technology and Applied Sciences (CKT‐UTAS) Navrongo Ghana; ^2^ Institute of Neuroscience, Third Affiliated Hospital Zhengzhou University Zhengzhou China; ^3^ Department of Global and International Health, School of Public Health University for Development Studies Tamale Ghana; ^4^ Department of Neurology TMSS Medical College and Hospital Thengamara Bogura Bangladesh

**Keywords:** cranial, irradiation, neurogenesis, neuroinflammation, neuroprotective, therapy

## Abstract

Cranial‐irradiation is associated with tissue damage resulting in neurocognitive impediments that adversely influence patient quality of life. Administration of radiation directly to the tumor may extend to the neighboring healthy tissues, which may induce acute to persistent oxidative stress, lessening neurogenesis, neuroinflammation, as well as vascular alterations, leading to neurocognitive sequelae as a result of decline in neuronal structural complexity as well as synaptic connections. Almost all the medications indicated in the treatment of irradiation‐triggered brain injury work via signaling pathways that are associated with lessening chronic oxidative stress, which is considered a consequence of the inflammatory response, reduction of edema, as well as microglia activation. Some agents have both preventative as well as therapeutic potential via the amalgamation of both neuroprotective and therapeutic mechanisms above. Thus, in this review, agents such as baicalein, troxerutin, epigallocatechin gallate, quercetin, melatonin, valproic acid, lithium, neurosteroid progesterone as well as minocycline have been implicated as neuroprotective agents for irradiation‐induced neurological deficits. Also, agents such as glucocorticoids, methylphenidate, vitamin E, bisdemethoxycurcumin, phosphodiesterases, edaravone, pioglitazone and fenofibrate, glutamate antagonists, human urinary kallidinogenase, bevacizumab, as well as hyperbaric oxygen have been implicated as therapeutic agents for irradiation‐induced neurological deficits. Furthermore, agents such as angiotensin‐converting enzyme, 3‐N‐butyl‐phthalide, stem cell therapy, sphingosine‐1‐phosphate, gangliosides, and neurotrophins have been implicated as combined potential neuroprotective and therapeutic agents for irradiation‐induced neurological deficits. The aim of this review is to elucidate the potential neuroprotective and therapeutic agents above and their mechanisms for irradiation‐induced neurological deficits after brain irradiation.

## Graphical abstract

In this review, agents such baicalein, troxerutin, epigallocatechin gallate, quercetin, melatonin, valproic acid, lithium, neurosteroid progesterone, as well as minocycline have been implicated as neuroprotective agents for irradiation‐induced neurological deficits. Also, agents such as glucocorticoids, methylphenidate, vitamin E, bisdemethoxycurcumin, phosphodiesterases, edaravone, pioglitazone and fenofibrate, glutamate antagonists, human urinary kallidinogenase, bevacizumab, as well as hyperbaric oxygen have been implicated as therapeutic agents for irradiation‐induced neurological deficits. Furthermore, agents such as angiotensin‐converting enzyme, 3‐N‐butyl‐phthalide, stem cell therapy, sphingosine‐1‐phosphate, gangliosides, and neurotrophins have been implicated as combined potential neuropreventative and therapeutic agents for irradiation‐induced neurological deficits.

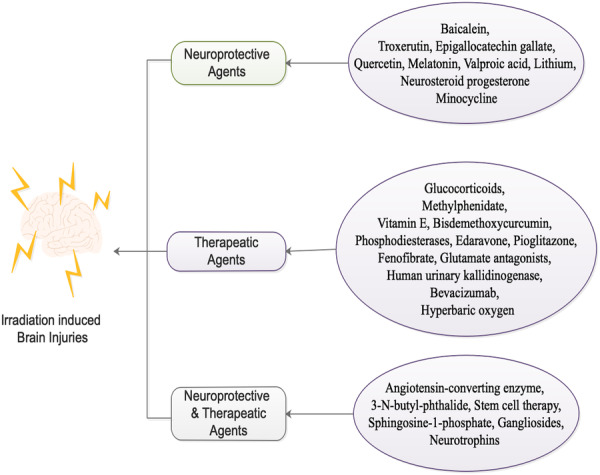

## 1 INTRODUCTION

Cranial irradiation or radiotherapy (IRT) is frequently accompanied by tissue damage resulting in neurocognitive impediments that adversely influence patients' quality of life.^1^, ^2^, ^3^ Exposed radiation to the tumor bed may extend to the bordering healthy tissues, which may trigger acute to persistent oxidative stress, reduction in neurogenesis, neuroinflammation, as well as vascular alterations, leading to neurocognitive sequelae as a result of reduction in neuronal structural complexity as well as synaptic connections.^1^, ^4^, ^5^, ^6^ The association between oxidative stress and IRT‐induced brain injury (RIBI) is well established, and it is also generally recognized that medications that decrease or inhibit chronic oxidative stress can avert or ameliorate cognitive impairment following RIBI ^7^, ^8^

Moreover, inflammatory responses have been implicated as a trigger of chronic oxidative stress, and immune cells, as well as stimulated microglia are capable of augmenting oxidative stress levels.^1^, ^2^, ^3^ Furthermore, IRT to microglial cells induced astrogliosis, leading to IRT‐induced edema.^9^ Additionally, in vitro studies demonstrated that stimulated microglia participate in triggering the disruption of neurogenesis via the expression of proinflammatory cytokines such as interleukin (IL)‐6, IL‐1β, and tumor necrosis factor‐a (TNF‐α), which stimulate a nonspecific reduction in cell survival and a selective reduction in neuronal differentiation among progenitor cells.^9^, ^10^, ^11^

It is reported that pharmacologic agents that exert antioxidant and anti‐inflammatory effects may mitigate the RIBI. Flavonoids have been implicated as neuroprotective agents following cranial‐IRT due to their antioxidant, anti‐inflammatory, as well as radio‐neuroprotective properties by modulating several intracellular and extracellular signaling pathways.^12^, ^13^ Non‐steroidal anti‐inflammatory drugs (NSAIDs) like indomethacin, were capable of restoring impaired neurogenesis triggered by cranial‐IRT via the blockade of inflammation.^14^ Furthermore, antiplatelet drugs such as cyclooxygenase (COX) inhibitors as well as adenosine diphosphate (ADP) receptor antagonists were also proven to be effective treatment for IRT‐induced neurological deficits.^14^ In addition, heparin and warfarin, which are anticoagulation drugs, were able to induce partial recovery of function in five of eight patients with cerebral radiation necrosis when they showed unresponsiveness to steroid therapy.^15^ Besides the above agents, several pharmacologic agents have been evaluated for symptomatic treatment of IRT‐induced neurological deficits.^7^, ^14^ Also, there are currently no approved preventive and therapeutic medications for IRT‐induced cognitive impairment in humans;^7^, ^14^ however, animal models of neuroprotection and therapeutic medications are promising. Thus, the aim of this review is to elucidate the potential neuroprotective and therapeutic agents and their mechanisms for IRT‐induced neurological deficits.

The “Boolean logic” was used to search for publications relevant to this topic in PubMed, PMC, Web of Science, and Google Scholar. No time restrictions were applied because this paper is a narrative review. Strict inclusion criteria were implemented, focusing exclusively on potential neuroprotective and therapeutic agents and their mechanisms for IRT‐induced neurological deficits. Notably, both in vitro and in vivo studies on the subject matter were included, while articles that described clinical studies were excluded because this review focused on mechanistic studies at the bench level.

## 2 IRRADIATION AND BRAIN INJURIES

RIBI has been categorized into acute, early delayed, as well as late delayed injury according to the time between the administration of IRT and the onset of clinical symptomatology.^14^, ^16^, ^17^ Acute RIBI transpires during and/or in days to weeks after IRT, while early delayed RIBI transpires between 6 and 12 weeks post‐IRT.^18^ Nevertheless, other researchers consider early delayed RIBI occurrence between 1 and 6 months.^18^, ^19^ It is worth noting that both RIBI above rarely transpire and typically resolve naturally or reversibly after short‐term treatment, although both of them can end up in life‐threatening reactions.^14^

On the other hand, late delayed RIBI, typically occurring 6 months post‐IRT, has been observed as irreversible and continuing unremittingly because of the pathogenesis.^14^, ^20^ Late delayed RIBI is not merely because of a decrease in the quantity of surviving clonogens of parenchymal or vascular target cell populations, but rather, it is an active activity that is also associated with stromal cells as well as impairment of the proliferation of neural precursors and continuous oxidative stress, resulting in tissue damage as well as functional deficits.^21^, ^22^, ^23^

Late delayed RIBI is often depicted with demyelination, white matter necrosis, reactive gliosis, vascular abnormalities, and perivascular edema, in a dose‐volume dependent severity on histopathological examinations of affected brains.^24^, ^25^ Also, late delayed RIBI was associated with a wide range of clinical symptoms like encephalopathy, syncope, seizure, memory loss, ataxia, and neuropathy, that severely impaired patients' quality of life.^24^, ^26^, ^27^

Generally, IRT is capable of triggering RIBI via either direct or indirect means so that neurogenesis and cognitive function are typically affected in the developing brain.^28^ Precisely, hippocampal neurogenesis and modifications in the microenvironment that result in changes in cognitive functions are appreciably affected by IRT.^28^, ^29^ Cranial‐IRT has been implicated in the triggering of complex modifications with at least four contributing factors such as damage to vessel structures, deletion of progenitor cells and mature oligodendrocytes, deletion of neural stem cells (NSCs) in the hippocampus, cerebellum, as well as cortex, and comprehensive changes in cytokine secretory levels.^30^, ^31^

## 3 NEUROPROTECTIVE AGENTS FOR RIBI

There are presently no clinically accepted preventive medications for radiation RIBI in humans;^7^, ^14^ however, animal models of neuroprotection medications are promising. The most widely investigated agents on RIBI are flavonoids, which are plant‐derived compounds with antioxidant, anti‐inflammatory, as well as radio‐neuro‐protective properties.^13^ These compounds and their metabolites are capable of crossing the blood‐brain barrier (BBB), which is composed of capillary endothelial cells, basement membrane, neuroglial membrane, as well as glial podocytes, which are projections of astrocytes.^32^, ^33^ They reach brain cells and decrease brain damage and relieve neurodegeneration that causes cognitive impairment.^34^

The neuroprotective machineries of flavonoids may include anti‐oxidation, anti‐apoptosis, anti‐neuroinflammation, as well as modulation of diverse intracellular and extracellular signaling targets.^12^ Pre‐irradiation treatment of male Wister rats with 5, 7‐dihydroxyflavone (DHF) led to augmented levels of acetylcholinesterase, malondialdehyde, β‐amyloid, and cysteine aspartic proteinase‐3 in the brains of rats following γ‐IRT.^35^ Notably, pre‐ and post‐treatment of male Wistar rats with 5,7‐dihydroxy‐8‐methoxy flavone (wogonin) following whole‐body γ‐IRT expressively decreased the concentration of TNF‐α, IL‐1β, and IL‐6, while mRNA and protein levels of heme oxygenase‐1 (HO‐1), glutathione (GSH), catalase (CAT), superoxide dismutase (SOD), glutathione peroxidase (GPx), and nuclear factor erythroid 2‐related factor 2 (Nrf2), were augmented.^12^, ^35^ This section highlights several widely studied flavonoids for mitigating RIBI. Meanwhile, other neuroprotective agents, including melatonin, lithium, the neurosteroid progesterone, and minocycline, have also been investigated for their potential to counteract RIBI. The neuroprotective agents for RIBI and their mechanisms of action were summarized in Table 1.

**Table 1:** The neuroprotective agents for IRT‐induced brain damage and the mechanisms of actions.

Neuroprotective agent	Mechanisms of action	Reference
Baicalein	It precisely protected cells against IRT and/or has the capacity to protect NPCs via means other than antioxidation because it protected NPCs from IRT‐stimulated cell death	[36]
	It decreased the loss of NPC proliferation and the generation of ROS in IRT hippocampi	[36]
	It decreased IRT‐induced hippocampal neuronal damage via the modulation of oxidative stress, as well as up‐modulation of BDNF‐p‐CREB signal transduction in both cell and animal experiments	[36]
Troxerutin	Pre‐IRT treatment of troxerutin offered neuroprotection against γ‐IRT‐stimulated neurotoxicity via stimulation of the PI3K/AKT/GSK3β/NrF2.	[47]
	Also, it induced phosphorylation state, which in turn triggered the scavenge free radicals' generation, alteration gene expression, as well as provided anti‐inflammatory effects in γ‐IRT rats	[47]
Epigallocatechin gallate	Pretreatment of irradiated rats with EGCG before γ‐IRT expressively downregulated augmented plasma concentrations of amyloid β, TNF‐α, as well as IL‐6, and reduced DA and serotonin levels	[49]
	EGCG treatment prior to IRT exposure protected against DNA damage as well as apoptosis in the hippocampus	[49]
	EGCG ameliorated the IRT‐stimulated decrease in antioxidants, such as GSH concentrations, and the activities of GPx, as well as GSR, in the hippocampus	[49]
	Pretreatment with EGCG prior to IRT inhibited inflammatory cytokine secretions as well as prevented augmentation of both hyper‐homocysteinaemia and amyloid β concentrations	[49]
	EGCG's blockade inflammation related to IRT‐stimulated hyper‐homocysteinaemia, as well as neuronal injury, was via its effective protection against the stimulation of immune response and inhibition of expression of TNF‐α, as well as oxidative stress	[49]
	EGCG also decreased p53, Bax, as well as caspases 3 and 9, but augmented Bcl‐2 expression following IRT	[49]
Quercetin	Pre‐IRT of male adult rat models with quercetin for 15 days augmented malondialdehyde concentrations as well as total antioxidant status in plasma and tissues after acute cranial‐IRT	[52]
	It appreciably decreased IRT‐stimulated neuronal degeneration as well as inflammatory infiltration, signifying its neuroprotective function after brain IRT exposure	[52]
	Quercetin pretreatment for 24 h before gamma IRT resulted in increased sustainability in primary cultured DRG neurons in concentration‐dependent manner	[52]
	Quercetin was able to decrease the secretion of the endoplasmic reticulum stress marker gene in IRT DRG neurons, as well as downregulated the secretion of TNF‐α	[52]
	It appreciably augmented the secretion of Tuj1 protein, which signifies neuronal revival, and reduced the apoptotic markers such as CHOP, JNK, as well as pJNK protein concentrations	[52, 54]
Melatonin	It can protect cells against hydroxyl radicals, which are the most common type of free radical, such as H_2_O_2_, NO, which are produced by immune cells, peroxynitrite anion, as well as singlet oxygen following IRT	[62, 63]
	Melatonin is capable of inducing genes and enzymes like Nrf2, SOD, GSH‐Px, GST, and GSR, all of which promote detoxification following IRT	[59, 64]
	Pretreatment with melatonin at distinctive doses before IRT may reduce oxidative damage via the modulation of SOD by decreasing O^2^^−^, as well as of GPx and CAT via reduction of H_2_O_2_	[65]
	Melatonin ameliorates the cranial‐IRT‐induced blockade of neurogenesis by decreasing oxidative stress	[66]
Valproic acid	Pre‐IRT with VPA protected against IRT brain damage in mouse hippocampus, as well as HT22, as compared to GL261 and D54 tumor models via the regulation of IRT‐stimulated pro‐apoptotic signaling.	[68]
	The attenuative effect of VPA correlated with a reduction in proapoptotic protein, such as Bax concentration, and augmented levels of Bcl‐2, an antiapoptotic protein, following IRT	[68]
	VPA treatment inhibited the phosphorylation of GSK3β (ser9) and stabilization of β‐catenin in normal and not in cancer cells following IRT	[75]
	GSK 3β blockade augmented the repair of DSB in DNA following IRT to normal HT22 but not in glioma cells, such as GL261 and D54, following VPA exposure	[68]
Lithium	Lithium pretreatment prior to IRT augmented cognitive performance of mice, as well as positively influences neurogenesis in the DG	[79, 80]
	Lithium was capable of reversing IRT‐induced NSPC dysfunction via the facilitation of neuronal progenitor proliferation as well as differentiation.	[83]
	Lithium treatment also prevented signs of IRT‐induced hypothalamus‐pituitary dysfunction without any obvious toxic effects	[81]
	Lithium treatment prevented the decrease of TSH as well as GH in the IRT mice, but did not reduce their body weight	[81]
	Lithium treatment also lessen BDNF concentrations, which is a sign of decreased hypothalamic/pituitary stress in IRT rats	[85]
	Lithium was capable of decreasing IRT‐stimulated DNA damage as well as annulled TP53‐associated IRT resistance in medulloblastomas, thus augmenting the anti‐tumor effects of IRT	[76, 88]
	Lithium was capable of preventing IRT‐stimulated acute inflammatory reactions 6 h after IRT, and the same phenomenon was perceived for cell death in the hippocampus	[81]
Neurosteroid progesterone	PROG was capable of decreasing both short as well as long‐term neurobehavioral or cognitive impairments in the IRT animals	[92]
	PROG was able to block IRT‐stimulated generation of reactive astrocytes as well as macrophage or microglial stimulatory machineries of the inflammatory cascade	[92]
	PROG was capable of decreasing apoptosis in the hippocampus as well as the cortex following IRT	[92]
	PROG was able to decrease cranial IRT‐stimulated damage to hippocampus and perirhinal cortex, resultin better memory in IRT mice	[92]
Minocycline	Its inhibitory effect on IRT‐induced hippocampal neuronal apoptosis expressively enhanced the cognitive performance of rats receiving WBRT	[94, 95]
	Its inhibitory effect on PARP‐1 was associated with oxidative stress‐induced neuronal death, inhibition of caspase‐1 and caspase‐3, as well as blockade of cytochrome c release from mitochondria, resulting in less neuronal apoptosis following IRT	[94, 96, 97]
	Minocycline had complex effects on DDR, such as blockade of ATM activation and γ‐H2AX secretion, neutral effect on p53 accumulation and 53BP1 foci stimulation, as well as augmented effect on IRT‐stimulated G2/M arrest in IRT HT22 cells	[94]
	It apparently blocked the upsurge of intracellular ROS quantities of IRT cells	[94]
	It protective machinery in IRT HT22 neurons was via AMPKα1‐facilitated autophagy following IRT	[94]
	It influenced brain cytokines such as IL‐10, IL‐15, and VEGF in mice receiving 1‐, 2‐, and 3‐Gy of IRT, which indicates it's immuno‐ and anti‐inflammatory effect, as well as its anti‐apoptosis and neuroprotective properties	[65]

*Note*: Refer to general abbreviation list for the meaning of abbreviations. All abbreviations and their full forms used in this paper can be found in the supplementary file.

### 3.1 Baicalein

Baicalein (5,6,7 trihydroxyflavone) has exhibited a wide range of therapeutic potentials in cardiovascular dysfunction, inflammation triggered by bacterial infections as well as cancer.^36^, ^37^ It has also proven to be beneficial in preventing spatial learning and memory impairment triggered by IRT.^36^ Thus, it may be an auspicious neuroprotective agent for IRT‐induced impairment of neurogenesis and its neurocognitive sequelae, as baicalein has the capacity to protect neural progenitor cells (NPCs) from IRT‐stimulated cell death.^36^ Also, baicalein pretreatment decreased the loss of NPC proliferation and the generation of reactive oxygen species (ROS), in IRT hippocampi.^36^

Furthermore, baicalein attenuated neuroinflammation within the subgranular zone (SGZ) as well as inhibited nitric oxide (NO) generation, inducible nitric oxide synthase (iNOS) protein, and nuclear factor kappa B (NF‐kB) activity in microglia.^38^ Baicalein augmented phosphorylated cAMP‐response element binding protein (pCREB) levels in the SGZ of the hippocampus in non‐IRT mice.^36^ Notably, it decreased IRT‐induced hippocampal neuronal damage via the modulation of oxidative stress with the up‐modulation of brain‐derived neurotrophic factor (BDNF)‐pCREB signal transduction in both cell and animal experiments.^36^ Thus, baicalein functions as an antioxidant to prevent loss of NPCs in the hippocampus and restores impaired neurogenesis by regulating BDNF‐pCREB signaling.

### 3.2 Troxerutin

Troxerutin (3′,4′,7‐Tris[O‐(2‐hydroxyethyl)] rutin) is a semi‐synthetic bioflavonoid derived from rutin.^39^ It is also known as vitamin P4, and it is gotten from coffee, tea, grains, fruits, and vegetables.^39^ Troxerutin has a high ability to cross the BBB as well as prevent rotenone‐stimulated retinal neurodegeneration.^40^ It also offers protection against oxidative stress, apoptosis, as well as inflammation.^41^, ^42^ Also, it was able to reduce neurotoxicity triggered by high cholesterol as well as excitotoxic neurodegeneration.^43^

Troxerutin has demonstrated to appreciably reduce proinflammatory cytokines such as interferon (INF)‐γ, extracellular signal‐related kinases (ERK)‐1/2, CCAAT/enhancer binding protein‐beta (C/EBP‐β) mRNA, and TNF‐α, with absolute mitigation of the oxidative stress condition, which was discovered by lessening ROS as well as carbonylated protein (CP) concentrations.^39^ It was further observed that the daily troxerutin administration reduced INFγ, IL‐1β, TNF‐α, and NF‐κB of activated B cells (p65) in the prolonged sciatic nerve constriction damage model.^44^ Also, ERK1/2 phosphorylation and activation were reversed with subsequent hindering of all signaling cascades as a result of a reduction in INFγ levels by troxerutin.^39^

Troxerutin triggered a neuroprotective effect by blocking the expression of a key neural cell death mediator called cyclin‐dependent kinase 1 (CDK1) in the hippocampus of dopamine (DA)‐treated rats.^45^ It is established that CDK1 typically blocks phosphorylation of protein phosphatase 1α at Thr320 and prevents it dephosphorylating, inactivating effect on mitogen‐activated protein kinase (MEK)‐1/2 as well as ERK1/2/C/EBP‐β activation.^45^ Thus, troxerutin inhibits the expression of inflammatory markers facilitated by C/EBP‐β and reverses memory impairment in DA‐treated mice through the blockade of MEK/ERK1/2 stimulation.^45^

Furthermore, troxerutin facilitated neuroprotective effects via reduction of advanced glycation end products (AGEs), ROS, and protein carbonyl levels as well as augmented phosphoinositide 3‐kinase/protein kinase B activation (PI3K/AKT), and apoptosis.^46^ Pre‐IRT treatment of troxerutin offered neuroprotection against γ‐IRT‐stimulated neurotoxicity via stimulation of the PI3K/AKT/glycogen synthase kinase 3 β/NrF2 pathway (PI3K/AKT/GSK3β/NrF2).^47^ Also, it induced a phosphorylation state which in turn triggered the scavenging of free radicals, alteration of gene expression, as well as provided anti‐inflammatory effects in γ‐IRT rats.^47^

### 3.3 Epigallocatechin gallate

Epigallocatechin gallate (EGCG) and its metabolites (theaflavins), are able to enter the brain parenchyma via the BBB, induce neuritogenesis and offer neuroprotective effects *in vitro* experiments.^48^ Pretreatment of irradiated rats with EGCG before γ‐IRT expressively downregulated augmented plasma concentrations of amyloid β, TNF‐α as well as IL‐6 and reduced DA and serotonin levels.^49^ Furthermore, EGCG treatment prior to IRT exposure protected against DNA damage as well as apoptosis in the hippocampus. EGCG ameliorated the IRT‐stimulated decrease in antioxidants, such as GSH concentrations, and the activities of GPx as well as glutathione reductase (GSR) in the hippocampus.^49^ It is well established that GSH is the substrate for GPx, and low GSH concentrations mean low GPx activity.^49^ However, pretreatment with EGCG expressively augmented the GSH concentrations as well as GPx activity in the hippocampus as well as prevented the loss of GSH and preserved the activities of GPx and GSR near the control values.^49^

It is worth noting that the mechanism underlying the antioxidative as well as neuroprotective effect of EGCG is via the GSH/GPx system.^49^ Also, cellular damage generated by ionizing radiation is often triggered by hydroxyl radicals generated by water radiolysis.^49^ Thus, the radioprotective effect of EGCG may be attributed to its capacity to scavenge these damaging radicals.^49^ Furthermore, pretreatment with EGCG prior to IRT inhibited inflammatory cytokine secretions as well as prevented augmentation of both hyper‐homocysteinaemia and amyloid β concentrations.^49^

EGCG attenuates inflammation associated with IRT‐induced hyperhomocysteinemia and neuronal injury by suppressing immune activation, inhibiting TNF‐α expression, and reducing oxidative stress.^49^ In addition, EGCG also decreased p53, B‐cell leukemia/lymphoma 2 protein (Bcl‐2) associated X (Bax), as well as caspases 3 and 9, but augmented Bcl‐2 expression.^49^ These molecular effects collectively indicate that EGCG can attenuate the severity of IRT‐stimulated damage by regulating inflammation and cell death in the hippocampus. Moreover, EGCG protected against neuronal injury as well as decreased monoamine concentrations via the protein kinase C α (PKCα) and ERK1/2 signaling pathways.^50^

Nevertheless, the fundamental instability of EGCG restricts its bioavailability as well as effectiveness.^51^ However, EGCG exhibited augmented stability when formulated as dual‐drug loaded nanoparticles (NPs) of EGCG/ascorbic acid (EGCG/AA NPs).^51^ In both in vitro and ex vivo, both EGCG and EGCG/AA NPs triggered tight junction distraction and opened the BBB. Thus, stabilization of EGCG in NP complexes as well as disruption of the BBB may result in higher remedial EGCG levels in the brain.^51^

### 3.4 Quercetin

Quercetin (3,3′,4′,5,7‐pentahydroxyflavone) is obtained from many vegetables as well as fruits.^52^ Its intake has been positively associated with improved inflammatory cardiovascular risk profiles, a benefit that may extend to other chronic inflammatory diseases.^53^ Recently, its neuroprotective effects against RIBI were also reported.^52^, ^54^ It was able to decrease lipopolysaccharide (LPS)‐stimulated NO release from a mouse neuroglia cell line and block cytokine production by astrocytes.^55^, ^56^ The stimulation of paraoxonase‐2 gene (PON2) by quercetin has been implicated in its anti‐inflammatory effects.^57^

Pre‐IRT of male adult rat models with quercetin for 15 days augmented malondialdehyde concentrations as well as total antioxidant status in plasma and tissues after acute cranial‐IRT.^52^ Also, quercetin appreciably decreased IRT‐stimulated neuronal degeneration as well as inflammatory infiltration, signifying its neuroprotective function after brain IRT exposure.^52^ Furthermore, quercetin pretreatment for 24 h before gamma IRT resulted in increased sustainability in primary cultured dorsal root ganglion (DRG) neurons in a concentration‐dependent manner.^52^

Quercetin was able to decrease the secretion of the endoplasmic reticulum stress marker gene in IRT DRG neurons and downregulate the secretion of TNF‐α.^12^ Additionally, it appreciably augmented the secretion of Tuj1 protein, which signifies neuronal survival, and reduced the apoptotic markers such as C/EBP‐homologous protein (CHOP), jun‐N‐terminal kinases (JNK), as well as pJNK protein concentrations.^52^, ^54^ Although no toxicity was reported,^52^, ^54^ quercetin has low bioavailability, and nanoparticles have been shown to augment its bioavailability.^58^

### 3.5 Melatonin

Melatonin (N‐acetyl‐5‐methoxytryptamine) is an altered tryptophan synthesized via the acetylation as well as methylation of serotonin.^59^ Melatonin is synthesized by the bone marrow, immune cells, brain, and the gut, although it is mainly secreted by the pineal gland.^60^ N1‐acetyl‐N2‐formyl‐5‐methoxykynuramine (AFMK) and N1‐acetyl‐5‐methoxykynuramine (AMK) are the key metabolites of melatonin, among which AFMK is the most abundant.^60^, ^61^ Melatonin and its metabolites have potent antioxidative as well as radioprotective properties.^59^, ^61^

Melatonin is a powerful scavenger of ROS/NO which triggers the release of free radicals and oxidative DNA damage.^62^, ^63^ It can protect cells against hydroxyl radicals, which are the most common type of free radical such as hydrogen peroxide (H_2_O_2_), NO which are produced by immune cells, peroxynitrite anion, as well as singlet oxygen following IRT.^62^, ^63^ Furthermore, melatonin is capable of inducing genes and enzymes like Nrf2, SOD, GSH‐Px, GST, and GSR, all of which promote detoxification following IRT.^59^, ^64^

Melatonin pretreatment expressively blocked the loss of proliferating as well as immature neurons in the DG via the eradication of oxidative stress.^65^, ^66^ Also, pretreatment with melatonin at distinctive doses before IRT may reduce oxidative damage via the modulation of SOD by decreasing O^2–^ as well as of GPx and CAT via reduction of H_2_O_2_.^65^ Furthermore, prolonged melatonin administration at doses of 10 mg/kg body weight for 7 days was capable of augmenting cell proliferation in the DG of maternally detached rats.^66^, ^67^ Thus, melatonin ameliorates cranial‐IRT‐induced blockade of neurogenesis by decreasing oxidative stress.^66^

### 3.6 Valproic acid

Valproic acid (VPA) is a short branched‐chain carboxylic acid for the treatment of seizures and depression.^68^, ^69^ The neuroprotective effect of VPA is via the blockade of histone deacetylase (HDAC) activity.^70^ VPA is able to block both class I and II HDACs, leading to hyperacetylation of histone H3 and H4.^71^ Also, VPA has been implicated in the Akt/GSK3β pathway, which is a key mechanism for its neuroprotective actions.^72^ It has further been implicated in the stimulation of alpha‐synuclein, heat shock protein 70 (HSP70), BDBF, as well as the regulation of the JNK pathway.^70^

VPA's ability to block HDAC results in interference in the repair of double‐strand breaks (DSB) in DNA breaks.^73^ VPA is capable of blocking cell growth in glioma cells via the stimulation of apoptosis as well as cell cycle arrest.^74^ Pre‐IRT with VPA protected against IRT brain damage in mouse hippocampus as well as hippocampus‐derived cells (HT22), as compared to GL261 and D54 tumor models.^68^ The selective protection of hippocampal neurons was a result of the regulation of IRT‐stimulated pro‐apoptotic signaling by VPA.^68^ Thus, VPA protection of normal cells from IRT damage was partly due to its mitigation of IRT‐stimulated apoptosis.

The attenuative effect of VPA correlated with a reduction in proapoptotic protein, such as Bax concentration, and augmented levels of Bcl‐2, an antiapoptotic protein following IRT.^68^ VPA treatment inhibited the phosphorylation of GSK3β (ser9) and stabilization of β‐catenin in normal and not in cancer cells following IRT.^75^ It is established that blockade of GSK3β, either with the small molecule inhibitor or knockdown, protected the hippocampal neurons from IRT‐induced apoptosis as well as augmented clonogenic survival.^75^ Also, GSK 3β blockade augmented the repair of DSB in DNA following IRT to normal HT22 but not in glioma cells such as GL261 and D54 following VPA exposure.^68^

### 3.7 Lithium

Lithium functions via the alteration of intracellular second messengers with consequent modulation of complex as well as interrelated intracellular enzyme cascades.^76^, ^77^, ^78^ It directly blocks Gsk3β, resulting in the enhancement of impaired cognition via different mechanisms the facilitation of neurogenesis, lessening of inflammation as well as apoptosis, which lead to long‐term potentiation as well as lessening of long‐term depression.^79^, ^80^ Lithium‐mediated blockade of GSK3β in microglia absolutely restores inflammation‐stimulated down‐modulation of antioxidant capacity in astrocytes and thus decreases cell death following oxidative stress.^81^, ^82^

Lithium has been used clinically for over 60 years, and it is still the treatment of choice for bipolar disorder.^76^, ^83^ Lithium has been implicated to be effective in the treatment of IRBI.^83^, ^84^ Lithium pretreatment prior to IRT augmented the cognitive performance of mice as well as positively influenced neurogenesis in the DG.^79^, ^80^ Lithium was capable of reversing IRT‐induced NSPC dysfunction via the facilitation of neuronal progenitor proliferation as well as differentiation.^83^ Lithium treatment also prevented signs of IRT‐induced hypothalamus‐pituitary dysfunction without any obvious toxic effects.^81^

Furthermore, Lithium treatment prevented the decrease of thyroid‐stimulating hormone (TSH) as well as GH in the IRT mice, but did not reduce their body weight.^81^ Lithium treatment also lessens BDNF concentrations, which is a sign of decreased hypothalamic/pituitary stress in IRT rats.^85^ Other studies have also affirmed the neuroprotective effects of lithium in cranial‐IRT models.^86^, ^87^ Blockade of stem or progenitor cell death as well as neuroinflammation, rescue of synaptic plasticity, and induction of stem and progenitor cell proliferation as well as neurogenesis are the protective properties of lithium.^76^, ^86^ Also, lithium does not protect cancer cells and thus functions as a radiosensitizer.^87^, ^88^

Lithium was capable of decreasing IRT‐stimulated DNA damage as well as annulled tumor protein 53 (TP53)‐associated IRT resistance in medulloblastomas, thus augmenting the anti‐tumor effects of IRT.^76^, ^88^ Lithium was able to prevent IRT‐stimulated cognitive deficits as well as signs of hypothalamus‐pituitary dysfunction without any obvious toxic effects.^81^ Thus, a combination of lithium with cranial‐IRT can effectively ameliorate the IRT‐induced brain injury as well as cognitive deficits.^81^ Also, lithium was capable of preventing IRT‐stimulated acute inflammatory reactions 6 h after IRT, and the same phenomenon was perceived for cell death in the hippocampus.^81^

### 3.8 Neurosteroid progesterone

The neurosteroid progesterone (PROG) is synthesized locally in the central nervous system (CNS) by glia and neurons.^89^, ^90^ PROG synthesis requires the transformation of cholesterol to pregnenolone by the cytochrome P450scc enzyme and then the transformation of pregnenolone to progesterone by the 3β‐hydroxysteroid dehydrogenases (3β‐HSD).^90^, ^91^ Also, progesterone is consecutively transformed into its neuroactive 5α‐reduced metabolites: 5α‐dihydroprogesterone (5α‐DHPROG) by 5α‐reductases; and 3α‐5α‐THPROG by 3α‐hydroxysteroid dehydrogenases (3α‐HSD).^90^, ^91^

Thus, the progesterone production in the CNS depends on its peripheral synthesis, brain uptake, as well as accumulation; its local synthesis; and its metabolism.^89^, ^91^ PROG has been implicated as a neuroprotective agent in several CNS injury models.^92^, ^93^ PROG was capable of decreasing both short as well as long‐term neurobehavioral or cognitive impairments in the IRT animals.^92^ Also, PROG was able to block IRT‐stimulated generation of reactive astrocytes as well as macrophage or microglial stimulatory machineries of the inflammatory cascade.^92^

Furthermore, PROG was capable of decreasing apoptosis in the hippocampus as well as the cortex following IRT.^92^ Additionally, PROG's ability to mitigate IRT‐stimulated injury machineries in the mice resulted in better functional and behavioral outcomes.^92^ Thus, PROG is a very beneficial treatment option for IR‐induced deficits in locomotor activity. Moreover, PROG was able to decrease cranial IRT‐stimulated damage to hippocampus and perirhinal cortex, resulting in better memory in IRT mice.^92^

### 3.9 Minocycline

Minocycline, a clinically approved second‐generation tetracycline antibiotic that can cross the BBB, significantly enhanced the cognitive performance of rats receiving whole‐brain irradiation therapy (WBRT), which was due to its inhibitory effect on IRT‐induced hippocampal neuronal apoptosis.^94^, ^95^ Minocycline's neuroprotective effects are via its inhibitory effect on Poly (ADP‐ribose) polymerase‐1 (PARP‐1), which is associated with oxidative stress‐induced neuronal death, inhibition of caspase‐1 and caspase‐3, as well as blockade of cytochrome c release from mitochondria, resulting in less neuronal apoptosis following IRT.^94^, ^96^, ^97^

Generally, tetracycline exerts neuroprotection via the blockade of autophagy in ischemic brain tissues, leading to the suppression of inflammatory cascades.^94^, ^98^ Minocycline had complex effects on DNA damage response (DDR), such as blockade of ataxia telangiectasia mutated (ATM) activation and gamma‐histone H2A variant X (γ‐H2AX) secretion, neutral effect on p53 accumulation and 53BP1 foci stimulation, as well as augmented effect on IRT‐stimulated G2/M arrest in IRT HT22 cells.^94^ Furthermore, minocycline apparently blocked the upsurge of intracellular ROS quantities of IRT cells.^94^

Nevertheless, minocycline did not accelerate IRT‐stimulated DNA damage repair, implying that the anti‐apoptotic effect of minocycline was related to DNA damage repair.^94^ Minocycline's protective machinery in IRT HT22 neurons was via adenosine monophosphate‐activated protein kinase 1 (AMPKα1)‐facilitated autophagy following IRT.^94^ Also, minocycline influenced brain cytokines such as IL‐10, IL‐15 and vascular endothelial growth factor (VEGF) in mice receiving 1‐, 2‐ and 3‐Gy of IRT, which indicates its immuno‐ and anti‐inflammatory effect, as well as its anti‐apoptosis and neuroprotective properties.^65^

## 4 THERAPEUTIC AGENTS FOR RIBI

Invariably, almost all the medications indicated in the treatment RIBI work via signaling pathways that are associated with neuroinflammation and microglia activation. Generally, chronic oxidative stress is considered to be a consequence of the inflammatory response.^1^, ^4^, ^99^ Also, immune cells and activated microglia can augment oxidative stress levels.^1^, ^4^ Furthermore, IRT to microglial cells stimulated astrogliosis and thus augments IRT‐induced edema.^9^ It was observed that recovery in memory‐deficient was a result of a decrease in the neuroinflammatory milieu, alterations in the repopulated microglia phenotype, as well as neuronal protein secretions.^1^, ^4^, ^99^

Precisely, repopulated microglia showed reduced secretion of complement mediator C5a receptor (C5aR) and phagocytic marker lysosomal‐associated membrane protein 1 (LAMP‐1).^99^ Also, microglia cognitive rescue correlated with an upsurge in synapsin‐1 and a decline in postsynaptic density protein‐95 (PSD‐95) protein concentrations.^99^ Microglia depletion after charged particle IRT absolutely prevented memory deficits measured after 90 days.^99^ Thus, temporary microglia depletion after IRT can act as a potent treatment countermeasure for the IRT‐stimulated memory deficits.^99^ The treatment of IRT‐induced edema typically involves the usage of corticosteroid medications because cerebral IRT necrosis is always accompanied by edema. In this section, we discussed potential therapeutic agents that either directly ameliorate RIBI‐associated neurological deficits by suppressing oxidative stress and neuroinflammation or target RIBI‐related symptoms and comorbidities, including cerebral edema, BBB disruption, fatigue, and radiation‐induced ischemia (Table 2).

**Table 2:** The therapeutic agents for IRT‐induced brain damage and the mechanisms of actions.

Therapeutic agents	Mechanisms of action	Reference
Glucocorticoids	They have polyvalent effectiveness of lessening lesions, alleviating symptoms, and taming their prognosis by neutralizing IRT‐induced vascular endothelial damage as well as the inflammatory cascade	[14, 104]
	GC suppresses the synthesis of numerous cytokines as well as chemokines, like GMCSF; IL‐1β, ‐4, ‐5, ‐8, and ‐10; as well as eotaxin and lipocortin 1, which are associated with the modulation of inflammatory reaction at the transcriptional stages following IRT	[105]
	GC also interacts with transcription factors like NF‐κB, AP‐1, p53, CRE‐binding protein, signal transducer, and the STAT family of activators of transcription, such as STAT3, STAT5, and others, indirectly inducing the actions of the latter on their own target genes following IRT	[105, 106]
	DXM, which is a GC, is a potent anti‐inflammatory drug that blocks the secretion of proinflammatory cytokines as well as adhesion molecules following IRT	[107]
	DXM is capable of blocking acute‐phase molecules like TNFA, IL1B, and ICAMI mRNA concentrations after brain IRT in several *in vivo* studies	[108, 109]
	DXM not only reduced leukocyte adhesion to vessel wall but also reverted the adhesion levels of ICAM1, E‐selectin, and P‐selectin microspheres to their baseline in IRT animals	[107, 108]
Methylphenidate	MPH elevates the synaptic concentration of DA and noradrenalin by inhibiting their reuptake, leading to an upsurge in the extracellular concentrations of these neurotransmitters in various brain regions following IRT	[114]
	It has demonstrated to be beneficial in boosting the cognitive dysfunction as well as depression in patients who received WBRT	[110, 111]
Vitamin E	Vit E expressively decreased the sternness of IRT‐induced brain damages, as well as augmented the activity of superoxide dismutase and catalase enzymes in the brain	[120]
	An obvious decrease in H3 acetylation at the BDNF gene promoter was detected post‐IRT, which was both HDAC1‐dependent as well as time‐dependent	[115]
Bisdemethoxycurcumin	BDMC augmented learning and memory, ameliorated brain edema, blocked astrocyte stimulation, as well as blocked oxidative stress after RIBI in rats	[8]
	Nrf2/HO‐1 signaling is triggered by several compounds linked to the immune response following RIBI	[131]
Phosphodiesterases	PDE inhibitors, which are antiplatelet nature, have exhibited robust protection against intravascular thrombosis after IRT	[7]
	Roflumilast, a PDE4 inhibitor, markedly blocked the generation of inflammatory mediators in macrophages via the stimulation of HO‐1 secretion as well as the blockade of NF‐κB, p38 MAPK, and JNK stimulation following IRT	[137]
Edaravone	Edaravone restored the differentiation as well as self‐renewal capability of NSCs even after they were exposed to IRT	[140]
	Edaravone‐treated patients had decreased edema area compared with that of patients from control group, signifying that it is capable of enhancing radiological improvement relevant to radio‐necrosis of the brain	[139]
	It has proven to be effective in preventing or mitigating the severity of RIBI	[14, 141, 142]
	It was proven to be effective in oxidative stress associated with RIBI	[14, 141, 142]
Pioglitazone & Fenofibrate	Pioglitazone activates PPARγ after binding, leading to the blockade of cytokines, adhesion molecules, as well as MMP's, and also prevents inflammatory cells from invading the central region of RIBI	[144, 148]
	Pioglitazone triggered the recovery of IRT‐induced cognitive impairment in rats	[142, 149]
	Fenofibrate augmented the number of hippocampal neurons as well as lessen microglial stimulation after WBRT in mice	[150]
Glutamate	Memantine normalized NMDA activity near physiologic levels despite high levels of Glu and thus could be favorable treatment option for IRT‐stimulated cognitive impairment if IRT‐induced ischemia occurs after fractional WBRT	[14, 156]
	Memantine inhibited the activities of Glu via binding to NMDA receptors and blocked the influx of Ca^2+^ preventing the disruption of synaptic plasticity following IRT.	[157, 158]
	Functional secretion of Ca^2+^‐permeable NMDARs in human GBM cell lines is correlated with a IRT response	[154]
	Ifenprodil, a GluN2B subunit‐specific NMDAR antagonist, triggered a reduced cell survival as well as cell migration leading a more distinct radiosensitizing effect on GBM as compared to MK80	[154]
	Stimulation of NMDARs and the successive phosphorylation of CREB enhanced the repair of IRT‐induced DSBs and NMDAR‐mediated downregulatory signaling via CREB was capable of protecting IRT‐induced cell damage in GBM	[154]
Human urinary kallidinogenase	HUK has been implicated in vasodilation of brain blood vessels, augmenting hemoglobin in the cerebral blood flow, as well as boosting glucose metabolism in ischemic brain tissues following IRT	[14, 165]
Bevacizumab	Bevacizumab was capable of alleviating the development of radionecrosis in numerous animal studies	[174, 175, 176]
	Bevacizumab was capable of relieving patients with IRT‐induced necrosis, which was established after T1‐weighted fluid‐attenuated inversion recovery signals and improvement in neurologic signs and symptoms of patients with RIBI	[14, 178]
	Blockade of VEGF at an early stage of IRT could decrease the development of IRT necrosis as well as vascular permeability	[171]
Hyperbaric Oxygen Treatment	Quantification of tissue O_2_ by means of TcpO_2_ exhibited that the O_2_‐tissue tension of IRT tissues recovers after only 8 regimens of HBOT	[179]
	High O_2_ gradient established between neighboring healthy tissues and irradiated tumors triggered angiogenesis, induced microvascularization, as well as neocollagenization	[179]

*Note*: Refer to general abbreviation list for the meaning of abbreviations.

### 4.1 Glucocorticoids

Glucocorticoids (GC) have pleiotropic influences on the brain.^100^, ^101^ GCs are essential for neuronal differentiation, integrity, growth, as well as synaptic and dendritic plasticity at the cellular level.^100^, ^101^ The cellular processes above aid in brain functions like motor control, decision‐making, learning and memory, reward‐based behavior, visual information processing, food intake, and energy modulation.^100^, ^102^, ^103^ GCs have been implicated in a broad range of therapies for RIBI. It is established that they have polyvalent effectiveness of lessening lesions, alleviating symptoms, and taming their prognosis by neutralizing IRT‐induced vascular endothelial damage as well as the inflammatory cascade.^14^, ^104^

GC suppresses the synthesis of numerous cytokines as well as chemokines, like granulocyte–macrophage colony‐stimulating factor (GMCSF), IL‐1β, ‐4, ‐5, ‐8, and ‐10, as well as eotaxin and lipocortin 1, which are associated with the modulation of inflammatory reaction at the transcriptional stages following IRT.^105^ GC also interacts with transcription factors like NF‐κB, activating protein 1 (AP‐1), p53, CRE‐binding protein, signal transducer, and the signal transducer and activator of transcription (STAT) family of activators of transcription, such as STAT3, STAT5, and others, indirectly inducing the actions of the latter on their own target genes following IRT.^105^, ^106^

Dexamethasone (DXM) is a potent anti‐inflammatory GC drug that blocks the secretion of proinflammatory cytokines as well as adhesion molecules following IRT.^107^ DXM is capable of blocking acute‐phase molecules like TNF‐a, IL‐1B, and intercellular adhesion molecule 1 (ICAM1) mRNA concentrations after brain IRT in several *in vivo* studies.^108^, ^109^ DXM not only reduced leukocyte adhesion to the vessel wall but also reverted the adhesion levels of ICAM1, E‐selectin, and P‐selectin microspheres to their baseline in IRT animals.^107^, ^108^ Therefore, DXM can alleviate radiation‐induced BBB breakdown.

### 4.2 Methylphenidate

Methylphenidate (MPH) is a psychostimulant and a DA reuptake inhibitor that augments the levels of DA in the frontal cortex in the CNS.^110^, ^111^ It is used in the treatment of conditions like depressive states, chronic fatigue, lethargy, narcolepsy, and disturbed senile behaviors.^110^, ^111^ Nonetheless, recent key satisfying use of MPH is for the management of narcolepsy as well as attention deficit hyperactivity disorder (ADHD) in children ^112^ such as selective attention, sustained attention as well as divided attention.^113^

MPH elevates the synaptic concentration of DA and noradrenalin by inhibiting their reuptake, leading to an upsurge in the extracellular concentrations of these neurotransmitters in various brain regions following IRT.^114^ Thus, MPH can be used to treat the fatigue that typically arises in patients receiving WBRT. Also, it demonstrated to be beneficial in boosting the cognitive dysfunction as well as depression in patients who received WBRT.^110^, ^111^ However, the side effects for MPH are anxiety as well as insomnia.

### 4.3 Vitamin E

Vitamin E (Vit E) is a lipid‐soluble antioxidant that decreases ROS.^115^ Vit E deficiency triggers degeneration of neurons, principally peripheral axons as well as posterior column neurons, which leads to peripheral neuropathy, ataxia, as well as skeletal myopathy.^116^, ^117^ It crosses the BBB and accumulates with time at therapeutic levels in the CNS.^115^ Vit E was able to prevent spatial learning deficits in the animal model of aging.^118^ Also, it boosted cognitive function in the animal model of Alzheimer's disease (AD) with repetitive concussive brain injury, as well as neuroprotection for several other types of brain insults.^119^

Furthermore, Vit E supplementation before traumatic brain injury (TBI) decreased oxidative stress as well as augmented learning and memory in numerous animal models.^115^, ^116^ Moreover, Vit E was capable of decreasing microscopic brain damage, stimulating nerve regeneration, as well as augmenting cognitive function in animal models following TBI.^116^, ^119^ Vit E has also proven to be a potential radioprotective drug for IRT‐induced neurological deficits.^120^ Vit E significantly decreased the IRT‐induced brain damage with the augmented activity of SOD and CAT enzymes in the brain.^120^

Vit E was capable of decreasing cognitive deficits triggered by the consumption of a diet high in saturated fat.^121^ BDNF is produced mainly by neurons located in the hippocampus, which is associated with functions such as learning and memory.^122^ Thus, BDNF augments the function as well as sustainability of select neuronal populations, and its role seems to be vital in the conservation of molecular processes essential for cognitive function.^122^, ^123^ Furthermore, BDNF stimulates neuronal excitability as well as facilitates synaptic function.^122^, ^123^ It is well established that oxidative stress may hinder cognitive function as well as neuroplasticity via the disruption of BDNF function.^123^

Vit E significantly stabilized concentrations of BDNF, which is crucial for conservation of synaptic plasticity as well as cognition. Also, the antioxidant capability of Vit E was associated with the regularization of antioxidant machineries such as SOD and sirtuin (SIR)‐2.^115^ Furthermore, an obvious decrease in H3 acetylation at the BDNF gene promoter was detected post‐IRT, which was both histone deacetylase 1 (HDAC1)‐dependent as well as time‐dependent. However, no substantial DNA methylation of the BDNF gene was identified after WBRT.^115^

### 4.4 Bisdemethoxycurcumin

Bisdemethoxycurcumin (BDMC), a demethoxy derivative of curcumin, is the most stable as well as potent curcuminoid.^8^, ^124^ Curcumin, a phenolic compound, is a derivative of Curcuma longa with numerous beneficial functions, such as anti‐oxidant, anti‐inflammatory, as well as neuroprotective properties.^125^ BDMC, which is founded on the curcumin matrix and produced by knocking out the methoxy group at the three positions on the bilateral benzene ring whilst preserving the four‐position hydroxyl group, demonstrates superior nuclear uptake, stability, polarity, hydrophilicity, as well as water solubility than curcumin.^126^ Thus, BDMC is more effective than curcumin.

The therapeutic mechanism of BDMC in the nervous system includes anti‐proliferative, anti‐inflammatory, and anti‐oxidant.^127^ BDMC was capable of protecting against 6‐hydroxydopamine hydrobromide‐stimulated brain lesions in rats.^128^ Also, BDMC was able to regulate adenosine 5′‐monophosphate‐activated protein kinase with the upregulation of SIR1, resulting in a decrease in oxidative stress in AD.^129^

ROS stimulates the expression of Nrf2 which binds to the anti‐oxidant response element located in the promoter region of its target gene.^130^ Nrf2 translocates to the nucleus, where it stimulates HO‐1 transcription to elicit its anti‐oxidant effects.^8^ Nrf2/HO‐1 signaling is triggered by several compounds linked to the immune response following RIBI.^131^ Thus, BDMC was capable of stimulating Nrf2/HO‐1 signaling after RIBI. Also, BDMC augmented learning and memory, ameliorated brain edema, blocked astrocyte stimulation, as well as blocked oxidative stress after RIBI in rats.^8^

### 4.5 Phosphodiesterases

Phosphodiesterases (PDEs) comprise 11 families such as PDE1‐PDE11 and they are responsible for the degradation of cyclic nucleotides.^132^, ^133^ The distributions of PDE subfamilies are distinct in diverse cells as well as tissues including the brain and they are associated with cognition, lipogenesis, apoptosis, proliferation, inflammation, as well as differentiation.^132^, ^134^ Amongst all the PDEs, the cAMP‐specific PDE4 is highly secreted in the brain.^132^ Thus, innovations in PDE4‐targeted treatment have exhibited promising responses in neuroinflammation‐associated diseases.^132^

Rolipram is a PDE4 inhibitor that has the ability to cross the BBB and was developed to ameliorate neuroinflammation.^132^ The blockade of PDE4 resulted in the elevation of intracellular cAMP levels and consequently alteration of inflammatory responses as well as preservation of immune balance.^135^ Moreover, PDE4 is a downstream regulator of β‐adrenoceptor as well as N‐methyl‐d‐aspartic acid receptor (NMDAR) mediated signaling pathway and also associated with 5‐hydroxytryptamine (5‐HT) receptor.^136^

Furthermore, roflumilast, another PDE4 inhibitor, markedly blocked the generation of inflammatory mediators in macrophages via the stimulation of HO‐1 secretion as well as the blockade of NF‐κB, p38 MAPK, and JNK stimulation following IRT.^137^ Noticeably, PDE inhibitors, which are antiplatelet nature, have exhibited robust protection against intravascular thrombosis after IRT.^7^ It is thus plausible that PDE4‐targeted treatment might ameliorate RIBI, and further studies are required in this direction.

### 4.6 Edaravone

Edaravone (3‐methyl‐1‐phenyl‐2‐pyrazolin‐5‐one), a new ROS scavenger and peroxisome proliferator‐activated receptor gamma (PPARγ) agonist, is also an antioxidant and oxidative stress inhibitor.^138^ It has been utilized in the treatment of several oxidative stress‐associated diseases, such as cerebral ischemia, by scavenging free radicals as well as inhibiting inflammatory reactions.^139^ Edaravone interacts with numerous free radicals, donates electrons, and ultimately transforms itself to the stable chemical called 2‐oxo‐3‐(phenylhydrazono)‐butanoic acid (OPB).^140^

This agent bestows neuroprotective effects in the brain by lessening the levels of iNOS following ischemia.^140^ On the one hand, edaravone inhibits endothelial injury as well as neuronal damage in brain ischemia. Edaravone protected cultured human NSCs from IRT‐induced cell death (RICD) and the protective effect more obvious in NSCs than brain tumor cells.^140^ On the other hand, edaravone restored the differentiation as well as self‐renewal capability of NSCs even after they were exposed to IRT.^140^ Thus, edaravone augmented the tolerance of NSCs to RIBI.

Edaravone‐treated patients had decreased edema area compared with that of patients in the control group, signifying that adding edaravone to the corticosteroid regimen can enhance radiological improvement relevant to radio‐necrosis of the brain.^139^ Thus, it has proven to be effective in preventing or mitigating the severity of RIBI.^14^, ^141^, ^142^ Also, it was proven to be effective in oxidative stress associated with RIBI.^14^, ^141^, ^142^ Nevertheless, some studies observed that it offers protection to brain tumors just as they offer protection to the normal brain so they have received not so much attention.^7^, ^14^

### 4.7 Pioglitazone and fenofibrate

Peroxisomal proliferator‐activated receptors (PPARs) are a nuclear hormone receptor family that are able to trigger neuroprotective as well as anti‐inflammatory pathways in the CNS.^143^

PPAR agonists inhibit proinflammatory cytokine secretion in both microglia as well as astrocytes and augment neuroprotection in animal models of stroke and neurodegenerative diseases.^143^ Pioglitazone, a thiazolidinedione drug as well as a synthetic agonist of PPARγ, has been demonstrated to be an effective anti‐inflammatory and neuroprotective agent in animal models of CNS injury.^144^, ^145^

The protective anti‐inflammatory influence of PPARγ was partially facilitated via transrepression of the redox‐modulatory transcription NF‐kB.^144^, ^146^, ^147^ Pioglitazone activates PPARγ after binding it, leading to the blockade of cytokines, adhesion molecules as well as matrix metalloproteinases (MMPs), and also prevents inflammatory cells from invading the central region of RIBI.^144^, ^148^ Thus, pioglitazone triggered the recovery of IRT‐induced cognitive impairment in rats.^142^, ^149^ Additionally, fenofibrate, an agonist of PPARα, augmented the number of hippocampal neurons and lessened microglial stimulation after WBRT in mice.^150^

### 4.8 Glutamate antagonists

Glutamate (Glu) is an excitatory neurotransmitter in the CNS, which is essential for normal synaptic transmission.^151^ Nonetheless, neurotoxic cascades, such as excitotoxicity as well as oxidative Glu toxicity, may occur when Glu release and uptake are not modulated.^152^ Thus, extracellular Glu must be eliminated rapidly to preserve Glu below toxic levels.^152^ The uptake of extracellular Glu is mediated by Glu transporters, which are also responsible for the long‐term preservation of low non‐toxic Glu levels.^153^ Glu is responsible for the collaboration of neurons and astrocytes as interdependent metabolic units.^151^

Residues Glu in the synaptic cleft after presynaptic release is eliminated predominantly by astrocytes via uptake.^151^ Glu‐triggered excitotoxicity is a major neurotoxic modality for many acute occurrences such as brain ischemia and trauma, as well as chronic neurodegenerative disorders such as amyotrophic lateral sclerosis.^151^ Glu activities are facilitated via metabotropic glutamate receptors (mGluRs) as well as ionotropic glutamate receptors (iGluRs).^154^ Also, the iGluRs are categorized into three subgroups, such as NMDAR, α‐amino‐3‐hydroxy‐5‐methyl‐4‐isoxazolepropionic acid (AMPA), as well as kainate receptors (KARs).^154^, ^155^

Memantine, an NMDAR antagonist, is capable of inhibiting ischemia‐stimulated NMDA excitation and has been demonstrated to be effective in vascular dementia.^14^ It is a low‐affinity voltage‐dependent noncompetitive glutamatergic NMDAR antagonist and it is used to treat cognition, behavior, mood, and physical functioning in AD.^156^ Memantine normalized NMDA activity near physiologic levels despite high levels of Glu.^156^ Consequently, it is assumed that it could be a favorable treatment option for IRT‐stimulated cognitive impairment if IRT‐induced ischemia occurs after fractional WBRT.^14^

Memantine inhibited the activities of Glu via binding to NMDARs and blocked the influx of Ca^2+^, preventing the disruption of synaptic plasticity following IRT.^157^, ^158^ Similarly, it is potentially neuroprotective in IRT‐induced apoptosis as well as oxidative stress in the CNS. Besides, memantine wielded radioprotective effects in adult patients in clinical trials.^157^, ^159^ However, there are no effective long‐term therapeutic or preventive schemes for IRT‐induced cognitive impairments in children or adolescents.^158^, ^160^ Further studies are needed in this direction.

Furthermore, functional secretion of Ca^2+^‐permeable NMDARs in human glioblastoma multiform (GBM) cell lines is correlated with an IRT response.^154^ Ifenprodil, a GluN2B subunit‐specific NMDAR antagonist, reduced cell survival and cell migration, leading to a more distinct radiosensitizing effect on GBM as compared to MK80.^154^ Also, stimulation of NMDARs and the successive phosphorylation of CREB enhanced the repair of IRT‐induced DSBs, and NMDAR‐mediated downregulatory signaling via CREB was capable of protecting IRT‐induced cell damage in GBM.^154^

### 4.9 Human urinary kallidinogenase

Human urinary kallidinogenase (HUK) is a glycoprotein extracted from human urine and capable of activating as well as modulating the kallikrein‐kinin system positively.^161^, ^162^ It is also capable of catalyzing the hydrolysis of low molecular weight kininogens to vasoactive kinins.^161^, ^163^ Furthermore, HUK treatment stimulated post‐ischemic angiogenesis as well as cerebral perfusion by triggering bradykinin B1 and B2 receptors.^161^, ^164^ Moreover, HUK offers protection via multiple mechanisms such as anti‐inflammation, anti‐apoptosis, promoting angiogenesis, as well as neurogenesis.^162^, ^164^

Mechanistically, HUK stimulated VEGF as well as apelin/APJ in ERK1/2 dependent way in middle cerebral artery occlusion (MCAO) rats as well as endothelial cell cultures.^164^ HUK potentially accelerated tube configuration as well as branches of vessels via up‐modulating ERK1/2‐apelin‐APJ‐VEGF pathway.^164^ Furthermore, tissue kallikrein/kinin, through the stimulation of kinin B2 receptor, facilitated angiogenesis via Akt‐GSK‐3β‐VEGF‐VEGFR‐2 as well as Akt‐eNOS‐NO signaling pathways.^162^ HUK has been implicated in vasodilation of brain blood vessels, augmenting hemoglobin in the cerebral blood flow, as well as boosting glucose metabolism in ischemic brain tissues following IRT.^14^, ^165^

Also, HUK was able to block Glu‐stimulated cell death as well as morphological modifications in cultured cortical neurons via the blockade of ROS generation.^166^ Furthermore, HUK attenuated nNOS activity, resulting in the blockade of Glu‐stimulated nitrosative stress via the modulation of intracellular signaling pathways such as B2R, ERK1/2, and NF‐kB, as well as up‐modulation of BDNF and Bcl‐2 secretion.^166^, ^167^, ^168^, ^169^ Moreover, HUK modulated ERK1/2 signaling cascades via Homer1b/c.^166^, ^170^ Further studies on the therapeutic effects of HUK on IRT‐induced neurological deficits are needed.

### 4.10 Bevacizumab

Bevacizumab, a monoclonal anti‐VEGF antibody, binds to VEGF and precludes it from attaching to receptors on the endothelial cell surface, thus reducing blood vessels as well as decreasing vascular permeability and edema.^171^, ^172^ Also, white matter surrounding necrotic areas has been discovered as the main VEGF up‐modulating site.^173^, ^174^ Moreover, augmented levels of VEGF in reactive astrocytes all over the core of necrotic tissue have been identified.^173^, ^174^ Also, bevacizumab was capable of alleviating the development of radionecrosis in numerous animal studies.^174^, ^175^, ^176^ Cranial‐IRT often triggers BBB disruption and endothelial cell injury, followed by tissue edema and hypoxia, which in turn triggers an upsurge in the secretion of hypoxia‐inducible factor (HIF)‐1a, which induces VEGF production.^172^, ^177^

VEGF is the most potent proangiogenic factor, and its high secretory levels trigger neovascularization with anomalous and fragile vessels, which facilitates brain edema, neuronal demyelination, as well as necrosis.^172^, ^177^ Thus, blocking VEGF at an early stage of IRT could decrease the development of IRT necrosis as well as vascular permeability.^171^ Also, bevacizumab was capable of relieving patients with IRT‐induced necrosis, which was established after T1‐weighted fluid‐attenuated inversion recovery signals and improvement in neurologic signs and symptoms of patients with RIBI.^14^, ^178^

### 4.11 Hyperbaric oxygen treatment

Hyperbaric oxygen treatment (HBOT) is capable of triggering physiological effects that augment plasma O_2_ transportation as well as better tissue accessibility.^179^, ^180^ Also, HBOT is proven to be able to restore the regional blood supply by reaching the goal of increasing parenchymal O_2_ concentration.^179^ The mechanism of action of HBOT was originally elucidated via the amalgamation of the solumetric as well as volumetric effects of breathing pure O_2_ inside a pressurized cabin.^179^ That is, the total of high dissolved 0_2_ tension in the plasma as well as its readiness to tissues.^179^

Furthermore, quantification of tissue 0_2_ by means of transcutaneous oximetry (TcpO_2_) exhibited that the 0_2_‐tissue tension of IRT tissues recovers after only 8 regimens of HBOT.^179^ A plateau was reached in the evolution curve, with TcpO_2_ values of 80‐85% compared to healthy non‐IRT tissues after this point. These values were sustained for at lowest of 3 years, without the persistent need for HBOT.^179^, ^181^ The high 0_2_ gradient established between neighboring healthy tissues and irradiated tumors triggered angiogenesis, induced microvascularization, as well as neocollagenization.^179^ HBOT has proven to be advantageous in pediatric patients with radionecrosis.^14^, ^182^, ^183^, ^184^

## 5 COMBINED POTENTIAL OF NEUROPROTECTIVE AND THERAPEUTIC AGENTS

It is worth noting that some agents have both neuroprotective as well as therapeutic potential via several distinctive mechanisms as well as signaling pathways (Table 3).

**Table 3:** The neuroprotective and therapeutic agents for IRT‐induced brain damage and the mechanisms of actions.

**Neuroprotective & therapeutic agent**	**Mechanisms of action**	**Reference**
Angiotensin‐converting enzyme	The effectiveness of renin‐angiotensin system blockers to prevent/ameliorate the severity of RIBI reveals blockade of an ongoing interaction between IRT and Ang II	[188]
	ACEI‐mediated neuroprotection against later RIBI in rat model of IRT‐induced injury to the optic nerve	[191]
	ACE inhibitors were able to alleviate radionecrosis by decreasing proangiogenic factors such as HIF‐1a and VEGF, CD31 via a decrease in Ang II secretion	[171]
	After tissue injury, AT2Rs are upregulated and Ang II, in amalgamation with other cytokines as well as growth factors, generates pro‐apoptotic, pro‐inflammatory, and pro‐oxidant effects, sustain long‐term tissue injury	[10, 188]
	Ang II stimulates PI3K as well as MAP kinase pathways to induce rapid signaling responses in the brain following IRT	[189]
	Ang II and IRT mediate biological effects of adhesion molecules, cytokines, and chemokines via the production of ROS, either by direct actions of the peptide, or indirectly via the stimulation of proinflammatory mediators that participate in ROS production	[185]
	Ramipril was capable of decreasing VEGF secretion, which also correlated with enhanced paralysis outcomes in IRT rats	[171, 192]
	Ramipril was able to decrease the incidence of IRT‐induced optic neuropathy, cognitive impairment, as well as IRT‐stimulated myelopathy	[191, 192, 193]
	Ramipril is able to alleviate the development of late or delayed effects of IRT after WBRT in doses above the necrotic threshold	[191]
3‐N‐butyl‐phthalide	NBP has a positive protective effect on oxidative cell damage, which makes it a potential neuroprotective agent following RIBI	[198]
	NBP is capable of reconstructing microcirculation, safeguarding mitochondrial function, blockade of oxidative stress, blockade of inflammatory responses, as well as blockade of neuronal apoptosis following IRT	[14, 199]
	BNP significantly improves the decrease in neuronal mitochondrial complex IV activity stimulated by low glucose and hypoxia, augmenting the actions of SOD and GSH‐Px as well as the content of MDA in mitochondria following IRT	[199]
Stem cell therapy	Stem cell therapy is a new strategy that could be used to prevent and treat IRBI	[201, 202]
	Grafted hMSCs have demonstrated to have neuroprotective potentials after cranial‐IRT	[202]
	Grafted hMSCs modulated the gene secretory profile of IRT brain, altered genetic pathways linked to inflammation, immune system, as well as cell motility, and decreased oxidative damage, as well as neuronal loss in brains of irradiated mice	[201, 202]
	Intranasally delivered hMSCs stimulated IRBI repair as well as enhanced neurological function	[201, 202]
	OPCs obtained from human pluripotent stem cells and transplantation into rat's forebrain were capable of remyelinate the IRT brain as well as rescuing the mice cognitive deficit	[201, 203]
	Selective blockade of autophagy in NSCs decreased RICD as well as caspase‐dependent apoptosis in the DG and cerebellum using mice with neural‐specific deletion of autophagy‐associated 7 gene, which is involved in autophagy induction as well as autophagosome formation and also decreased the levels of pro‐inflammatory cytokines	[201, 204]
	The usage of NSCs replacement forms a novel substitute to combat IRT‐induced cognitive decline because IRT triggers progressive depletion of NSCs	[201, 204]
	NSCs partially restored cognitive function into rodent brains after WBRT	[205, 206]
	The majority of slowly dividing NSCs survive a modest dose of IRT and enter the cell cycle to regenerate the irradiated neurogenic niche	[201, 207]
	Transcriptomic evaluation of NSCs obtained from the V‐SVZ zone of 2‐month‐old mice showed that genes upregulated after WBRT are mainly related to cell cycle, DNA/RNA processes, translation, as well as ribosomal activity	[201, 208]
Sphingosine‐1‐phosphate	Fingolimod, S1PR modulator (FTY720), has been implicated as a treatment and prevention of IRT‐stimulated cognitive dysfunction	[209]
	FTY720 was able to partly rescue IRT‐stimulated blockade of neurogenesis in mice after a single dose of 6‐Gy X‐ray IRT	[209]
	FTY720 was able to mitigate proinflammatory cytokines like IL‐1β, IL‐6, and TNF‐α after LPS‐induction of primary microglial cells following IRT	[209]
Gangliosides	Binding of GM1 to the TrkA facilitates receptor dimerization and activation, as well as downregulatory consequences like neurite outgrowth and neuroprotection and therapy following IRT	[219, 220]
Neurotrophins	Several *in vivo* and *in vitro* studies have exhibited that neurotrophic factors are neuroprotective and therapeutic in IRT‐induced neuropathy	[223, 224]
	IRT with 2‐Gy and 8‐Gy blocked the dephosphorylation of NFATc4/3 as well as its translocation	[223, 224]
	Stimulation of NFATc4/3 dephosphorylation via BDNF relieved IRT‐stimulated neurite outgrowth inhibition	[223, 224]
	DHF triggered TrkB and its downstream signaling pathways in the hippocampus after cranial‐IRT and augmented cognitive functions, particularly the hippocampal‐dependent functions	[228]
	DHF‐mediated protection of hippocampal functions after cranial‐IRT augmented long‐term survival of newborn neurons and well‐preserved synaptic structures in the hippocampal DG	[228]
	DHF treatment regularized many IRT‐induced behavioral defects, such as anxiety, as well as fear memory, working spatial memory, contextual memory, and spatial learning	[228]

*Note*: Refer to general abbreviation list for the meaning of abbreviations.

### 5.1 Angiotensin‐converting enzyme

Angiotensin‐converting enzyme (ACE) inhibitors are medications used for the treatment of hypertension and heart failure.^171^ Mechanistically, all enzymes required for consequent processing of angiotensin (Ang) I into active peptides such as ACE for Ang II, ACE2 and neprilysin for Ang I‐VII, as well as aminopeptidases for Ang III and IV are present in the CNS.^185^ Also, angiotensinogen is secreted widely in brain tissue.^186^ It is worth noting that Ang II is a potent vasopressor that functions by binding to G‐protein receptors such as Ang II type 1 receptor (AT1R) and Ang II receptor type 2 (AT2R), which are widely distributed in the brain and predominantly dense in the hippocampus.^10^, ^187^

It was observed that after tissue injury, AT2Rs are upregulated and Ang II, in combination with other cytokines as well as growth factors, generates pro‐apoptotic, pro‐inflammatory, and pro‐oxidant effects, sustaining long‐term tissue injury.^10^, ^188^ Ang II stimulates PI3K as well as MAPK pathways to induce rapid signaling responses in the brain following IRT.^189^ Ang II is a potent inflammatory peptide, mediating the secretion of proinflammatory mediators such as adhesion molecules, cytokines, and chemokines via the stimulation of the redox‐regulated transcription factors like AP‐1 and NF‐κB.^190^

Also, Ang II and IRT mediate their biological effects via the production of ROS, either by direct actions of the peptide, or indirectly via the stimulation of proinflammatory mediators that participate in ROS production.^185^ The effectiveness of renin‐angiotensin system blockers to prevent/ameliorate the severity of RIBI reveals blockade of an ongoing interaction between IRT and Ang II.^188^ Besides, ACE inhibitors mediated neuroprotection against later RIBI in the rat model of IRT‐induced injury to the optic nerve.^191^ Also, ACE inhibitors were able to alleviate radionecrosis by decreasing proangiogenic factors such as HIF‐1a and VEGF, CD31 via a decrease in Ang II secretion.^171^

Ramipril, one of the ACE inhibitors, was capable of decreasing VEGF secretion, which also correlated with enhanced paralysis outcomes in IRT rats.^171^, ^192^ Also, ramipril was able to decrease the incidence of IRT‐induced optic neuropathy, cognitive impairment, as well as IRT‐stimulated myelopathy.^191^, ^192^, ^193^ Ramipril has the ability to cross the BBB and thus influences the brain renin‐angiotensin system directly.^191^, ^192^, ^193^ Thus, ramipril is able to alleviate the development of late or delayed effects of IRT after WBRT in doses above the necrotic threshold.^191^ Furthermore, ramipril produced anti‐inflammatory/anti‐oxidant effects by antagonizing the actions of IL‐6, TNF‐α, monocyte chemoattractant protein‐1 (MCP‐1), as well as other cytokines, without influencing the quantities of activated microglia.^10^, ^194^

### 5.2 3‐N‐butyl‐phthalide

3‐N‐butyl‐phthalide (NBP) is a novel and powerful natural free radical scavenger, which is extracted from the seeds of Apium.^195^, ^196^ It is worth noting that both *in vivo* and *in vitro* studies have shown that NBP has therapeutic value in ischemic stroke, vascular dementia, AD, and parkinson's disease (PD).^196^, ^197^ Also, it enhances angiogenesis via up‐regulation of VEGF and HIF‐1a secretion, and protects endothelial cells against oxidative/nitrosative stress, mitochondrial damage as well as consequent cell death.^198^

Furthermore, NBP has a positive protective effect on oxidative cell damage, making it a potential neuroprotective agent following RIBI.^198^ Moreover, NBP, is capable of reconstructing microcirculation, safeguarding mitochondrial function, blockade of oxidative stress, blockade of inflammatory responses, as well as blockade of neuronal apoptosis following IRT.^14^, ^199^ NBP also has anti‐platelet aggregation, anti‐thrombosis, as well as anti‐atherosclerosis effects.^200^ In the mitochondrial membrane, NBP is able to augment the actions of Na^+^‐K^+^ ATPase and Ca^2+^‐ATPase.^199^

In addition, NBP significantly improves the decrease in neuronal mitochondrial complex IV activity stimulated by low glucose and hypoxia, augmenting the actions of SOD and GSH‐Px as well as the content of malondialdehyde (MDA) in mitochondria following IRT.^199^ Furthermore, NBP was able to reduce secretion of Bax, p‑JNK and p‑p38 MAPK, a reduced apoptotic rate as well as augmented Bcl‑2 secretion in cerebral infarction.^199^, ^200^ Further studies on the therapeutic effects of NBP on IRT‐induced neurological deficits are needed.

### 5.3 Stem cell therapy

Stem cell therapy is a new strategy that could be used to prevent and treat IRBI.^201^, ^202^ Human mesenchymal stem cells (hMSCs), have demonstrated to have neuroprotective potentials after cranial‐IRT.^202^ Grafted hMSCs modulated the gene secretory profile of IRT brain, altered genetic pathways linked to inflammation, immune system, as well as cell motility, and decreased oxidative damage as well as neuronal loss in brains of irradiated mice.^201^, ^202^ Also, intranasally delivered hMSCs stimulated IRBI repair as well as enhanced neurological function.^201^, ^202^ Thus, hMSCs could be used as a non‐invasive therapy to prevent neurological complications of IRT.^202^

Oligodendrocyte progenitors (OPCs), obtained from human pluripotent stem cells and transplantation into rats' forebrain, were capable of remyelinating the IRT brain and rescuing their cognitive deficits.^201^, ^203^ Moreover, recovery from motor deficits required a concurrent OPCs graft to the cerebellum.^201^, ^203^ Selective blockade of autophagy in NSCs decreased RICD as well as caspase‐dependent apoptosis in the DG and cerebellum using mice with neural‐specific deletion of the autophagy‐associated 7 gene, which is involved in autophagy induction as well as autophagosome formation. Also, this machinery decreased the levels of pro‐inflammatory cytokines.^201^, ^204^ Thus, autophagy might be another potential target for preventing RICD and its associated long‐term effects.

Furthermore, as IRT triggers progressive depletion of NSCs, the usage of NSCs replacement forms a novel substitute to combat IRT‐induced cognitive decline.^201^, ^204^ Moreover, NSCs partially restored cognitive function in rodent brains after WBRT.^205^, ^206^ Also, the NSCs not only differentiate into neurons, but also oligodendrocytes, astrocytes as well as endothelial cells, and are capable of changing the hippocampal microenvironment.^14^, ^205^ Furthermore, the majority of slowly dividing NSCs survive a modest dose of IRT and enter the cell cycle to regenerate the irradiated neurogenic niche.^201^, ^207^ Moreover, transcriptomic evaluation of NSCs obtained from the V‐SVZ zone of 2‐month‐old mice showed that genes upregulated after WBRT are mainly related to cell cycle, DNA/RNA processes, translation, as well as ribosomal activity.^201^, ^208^

### 5.4 Sphingosine‐1‐phosphate

Sphingosine‐1‐phosphate (S1P) is a signaling sphingolipid, also referred to as a lysosphingolipid, and is primarily identified for its immunosuppressive role by inhibiting the egress of lymphocytes from lymph nodes.^65^, ^209^, ^210^ S1P is able to stimulate the proliferation of cultured hippocampal precursors via the stimulation of MAPK pathways, and enhance NPC migration to the region of injury in an infarcted brain.^211^, ^212^ Fingolimod, sphingosine‐1‐phosphate receptor (S1PR) modulator (FTY720), has been implicated as a treatment and prevention of IRT‐stimulated cognitive dysfunction.^209^

S1PRs are secreted by several cells of the CNS, except S1PR4, and they are essential in neuronal development as well as angiogenesis.^209^ FTY720 is able to cross the BBB due to its lipophilic nature, and it exhibited positive effects on embryonic as well as adult neurogenesis. Furthermore, treatment with FTY720 increases proliferation and migration of embryonic NSCs in vitro as well as differentiation toward protoplasmic astrocytes.^213^ FTY720 was able to trigger the generation of NPCs in the hippocampus of healthy mice, resulting in the enhancement of memory as well as learning abilities.^214^, ^215^

Notably, FTY720 is able to enhance neuroprotection as well as decrease excitotoxicity and microglial stimulation in the CNS.^216^ Furthermore, FTY720 was able to partly rescue IRT‐stimulated blockade of neurogenesis in mice after a single dose of 6‐Gy X‐ray IRT.^209^ Moreover, FTY720 is able to target four of the five S1PR such as S1PR1, S1PR3, S1PR4, S1PR5 after phosphorylation in vivo.^209^ Additionally, FTY720 is able to mitigate proinflammatory cytokines like IL‐1β, IL‐6, and TNF‐α after LPS‐induction of primary microglial cells following IRT.^209^

### 5.5 Gangliosides

Gangliosides (GM1 & GM2) are glycosphingolipids comprising a ceramide lipid tail attached via glycosidic linkage to a glycan headgroup hosting one or more sialic acid residues.^217^, ^218^ They are the principal transporters of sialic acid, a terminal sugar responsible for cell‐cell as well as pathogen‐cell interactions.^217^, ^218^ They facilitate the recovery of nerve function in the lesioned brain, spinal cord, as well as peripheral nerve.^217^ Mechanistically, binding of GM1 to the tropomyosin‐receptor kinase (Trk)‐A facilitates receptor dimerization and activation, as well as downregulatory consequences like neurite outgrowth and neuroprotection and therapy following IRT.^219^, ^220^

Also, GM1 modulates GluR2‐AMPA receptor (AMPAR), an ionotropic Glu receptor for memory as well as learning processes.^221^, ^222^ Furthermore, focal production of GM1 via NEU3 was associated with neuronal polarity and the initiation of axons from multiple neurites in hippocampal cultures.^217^, ^219^ Moreover, GM1 was associated with focal enhancement of TrkA activity, blockade of RhoA signaling, as well as local actin depolymerization.^217^, ^219^ Studies on the therapeutic effects of GM1 & GM2 on IRT‐induced neurological deficits are needed.

### 5.6 Neurotrophins

Neurotrophins (NT) have been implicated in the facilitation of neuronal survival, via the modulation of neuronal development and function, such as synapse formation as well as synaptic plasticity.^223^ Four distinctive neurotrophins such as nerve growth factor (NGF), BDNF, NT‐3 and NT‐4 are expressed in mammals.^223^ Specifically, BDNF is capable of modulating neural circuit development as well as neuroplasticity.^224^, ^225^ Also, the up‐modulation of BDNF triggers morphological plasticity, such as modified dendritic fields as well as augmented axonal branching.^223^, ^224^, ^226^

Furthermore, BDNF was able to induce increased intracellular Ca^2+^ concentrations, which facilitated nuclear factor of activated T‐cells (NFATc)‐4/3 dephosphorylation, promoting neurite outgrowth.^223^, ^227^ BDNF binds at high affinity to TrkB and stimulates its downstream signaling pathways like the MAPK and PI3K pathways.^228^ Interesting, DHF has recently been recognized as a TrkB agonist,^229^ and notably, DHF was capable of crossing the BBB and expressly triggered the TrkB receptor in the brain, resulting in long‐lasting stimulation of BDNF signaling pathways.^229^, ^230^ Moreover, DHF exhibited antioxidant as well as anti‐inflammatory effects *in vitro* after oxidative insults or LPS‐induced injury, in addition to TrkB activation.^231^ Several in vivo and in vitro studies have exhibited that neurotrophic factors are neuroprotective and therapeutic in IRT‐induced neuropathy. Moreover, it was reported that IRT with 2‐Gy and 8‐Gy blocked the dephosphorylation of NFATc4/3 as well as its translocation.^223^, ^224^ However, the stimulation of NFATc4/3 dephosphorylation via BDNF relieved IRT‐stimulated neurite outgrowth inhibition.^223^, ^224^

DHF triggered TrkB and its downstream signaling pathways in the hippocampus after IRT and augmented cognitive functions, particularly the hippocampal‐dependent functions.^228^ Furthermore, DHF‐mediated protection of hippocampal functions after IRT augmented long‐term survival of newborn neurons and well‐preserved synaptic structures in the hippocampal DG.^228^ Moreover, DHF treatment regularized many IRT‐induced behavioral defects, such as anxiety, fear memory, working spatial memory, contextual memory, and spatial learning.^228^

## 6 CONCLUSION

Potential neuroprotective agents work via machineries such as anti‐oxidation, anti‐apoptosis, anti‐neuroinflammation, as well as modulation of diverse intracellular and extracellular signaling targets. In variably, almost all the medications indicated in the treatment RIBI work via signaling pathways that are associated with the reduction of chronic oxidative stress, which is considered a consequence of inflammatory response, reduction of edema, as well as microglia activation. It is worth noting that some agents have both preventative as well as therapeutic potential via the amalgamation of both neuroprotective and therapeutic mechanisms above.

## AUTHOR CONTRIBUTIONS

Seidu A. Richard contributed to the study concept and design, literature search and selection, and manuscript preparation. Seidu A. Richard, Vivian Kapio Abem, and Sagor Kumar Roy contributed to manuscript editing. All authors read and approved the final manuscript.

## CONFLICT OF INTEREST STATEMENT

The authors declare no conflicts of interest.

## ETHICS STATEMENT

Not applicable.

## Supporting information

Supporting information

## Data Availability

Not applicable as no new data were generated in this paper.
