# Supplementary material for: Potential neuroprotective and therapeutic agents and their mechanisms for irradiation‐induced brain injury
Source: Ibrain. 2026 Jan 15;12(1):96–122. doi: 10.1002/ibra.70011 (PMC13097531; doi:10.1002/ibra.70011)
Supplement: Supplementary file 1 — Supporting information [file IBRA-12-96-s001.docx]

*5, 7-dihydroxyflavone = DHF, 5-hydroxytryptamine = 5-HT, 2-oxo-3-(phenylhydrazono)-butanoic acid = OPB, 3-N-butyl-phthalide = NBP, Adenosine diphosphate = ADP, Advanced glycation end products = AGEs, Ataxia telangiectasia mutated = ATM, Adenosine monophosphate-activated protein kinase 1 = AMPKα1, Activating protein 1= AP-1, Attention deficit hyperactivity disorder = ADHD, α-amino-3-hydroxy-5-methyl-4-isoxazolepropionic acid = AMPA, Angiotensin-converting enzyme = ACE, Baicalein = 5,6,7 trihydroxyflavone, Brain-derived neurotrophic factor = BDNF, Phosphorylated-cyclic-AMP response element-binding protein = p-CREB, Blood brain barrier = BBB, B-cell leukemia/lymphoma 2 protein = Bcl-2, Bcl-2 associated X = Bax, Bisdemethoxycurcumin = BDMC, Catalase = CAT, CCAAT/enhancer binding protein-beta = C/EBP‐β, C/EBP-homologous protein = CHOP, Cyclin-dependent kinase 1 = CDK1, Central nervous system = CNS, Cranial irradiation or radiotherapy = IRT, Dopamine = DA, Dorsal root ganglion = DRG, Double strand breaks = DSB, DNA damage response = DDR, Dexamethasone = DXM, Extracellular signal-related kinases = ERK, Epigallocatechin gallate = EGCG, Edaravone = 3-methyl-1-phenyl-2-pyrazolin-5-one, Glutathione = GSH, Glutathione peroxidase = GPx, Glutathione reductase = GSR, Glycogen synthase kinase 3 β = GSK3β, gamma-histone H2A variant X = γ-H2AX, Glucocorticoids = GC, Granulocyte–macrophage colony-stimulating factor = GMCSF, Glutamate = Glu, Glioblastoma multiform = GBM, Gangliosides = GM1 & GM2, Heme oxygenase-1 = HO-1, Hydrogen peroxide = H2O2, Histone deacetylase = HDAC, Hippocampus-derived cells = HT22, Hydroxysteroid dehydrogenases = HSD, Human urinary kallidinogenase = HUK, Hypoxia-inducible factor = HIF, Hyperbaric oxygen treatment = HBOT, Human mesenchymal stem cells = hMSCs, Interleukin = IL, IRT-induced brain injury = RIBI, Inducible nitric oxide synthase = iNOS, Interferon = INF, Intercellular adhesion molecule 1 = ICAM1, IRT-induced cell death = RICD, Ionotropic glutamate receptors = iGluRs, Jun-N-terminal kinases = JNK, Kainate receptors = KARs, Lysosomal-associated membrane protein 1 = LAMP-1, Lipopolysaccharide = LPS, Mitogen‐activated protein kinase = MEK, Melatonin = N-acetyl-5-methoxytryptamine, Methylphenidate = MPH, Matrix metalloproteinases = MMP’s, Metabotropic glutamate receptors = mGluRs, Middle cerebral artery occlusion = MCAO, Monocyte chemoattractant protein-1 = MCP-1, Malondialdehyde = MDA, Nuclear factor erythroid 2-related factor 2 = Nrf2, Neural progenitor cells = NPCs, Nitric oxide = NO, Nuclear factor kappa B = NF-kB, Nanoparticles = NPs, N1-acetyl-N2-formyl-5-methoxykynuramine = AFMK, N1-acetyl-5-methoxykynuramine = AMK, Neurosteroid progesterone = PROG, N-methyl-D-aspartic acid receptor = NMDAR, N-methyl-D-aspartate = NMDA, Neural stem cells = NSCs, Neurotrophins = NT, Nerve growth factor = NGF, Nuclear factor of activated T-cells = NFATc, Oligodendrocyte progenitors = OPCs, Phosphorylated cAMP-response element binding protein = pCREB, Phosphoinositide 3‐kinase/protein kinase B activation = PI3K/AKT, Paraoxonase-2 gene = PON2, Poly (ADP-ribose) polymerase-1 = PARP-1, Phosphodiesterases = PDEs, Peroxisome proliferator-activated receptor gamma = PPAR, Parkinson's disease = PD, Quercetin = 3,3′,4′,5,7-pentahydroxyflavone, Reactive oxygen species = ROS, Superoxide dismutase = SOD, Subgranular zone = SGZ, Signal transducer and activator of transcription = STAT, Sirtuin = SIR, Sphingosine-1-phosphate = S1P, Sphingosine-1-phosphate receptor = S1PR, S1PR modulator = FTY720, Tumor necrosis factor-a = TNF-a, Troxerutin = 3′,4′,7-Tris[O-(2-hydroxyethyl)] rutin, Thyroid-stimulating hormone = TSH, Tumor protein 53 = TP53, Traumatic brain injury = TBI, Transcutaneous oximetry = TcpO2, Tropomyosin receptor kinase B = TrkB, Valproic acid = VPA, Vascular endothelial growth factor = VEGF, Vitamin E = Vit E, Whole brain irradiation therapy = WBRT.*
